# Internalized Functional DNA Aptamers as Alternative Cancer Therapies

**DOI:** 10.3389/fphar.2020.01115

**Published:** 2020-07-24

**Authors:** Morgan L. Marshall, Kylie M. Wagstaff

**Affiliations:** Biomedicine Discovery Institute, Monash University, Clayton, VIC, Australia

**Keywords:** DNA-aptamer, tumor-specific, precision medicine, intracellular targeting, nanoparticle-free

## Abstract

Despite major advances, cancer remains one of the largest burdens of disease worldwide. One reason behind this is that killing tumor cells without affecting healthy surrounding tissue remains a largely elusive prospect, despite the widespread availability of cytotoxic chemotherapeutic agents. To meet these modern healthcare requirements, it is essential to develop precision therapeutics that minimise off-target side-effects for various cancer types. To this end, highly specific molecular targeting agents against cancer are of great interest. These agents may work by targeting intracellular signalling pathways following receptor binding, or *via* internalization and targeting to specific subcellular compartments. DNA aptamers represent a promising molecular tool in this arena that can be used for both specific cell surface targeting and subsequent internalization and can also elicit a functional effect upon internalization. This review examines various cancer targeting cell-internalizing aptamers, with a particular focus towards functional aptamers that do not require additional conjugation to nanoparticles or small molecules to elicit a biological response. With a deeper understanding and precise exploitation of cancer specific molecular pathways, functional intracellular DNA aptamers may be a powerful step towards more widespread development of precision therapeutics.

## Introduction

Cancer is the second most prevalent non-communicable disease worldwide, which accounted for more than 9.6 million deaths in 2018 (Ferlay et al., 2019). Despite enhanced public health attention towards early diagnosis and commitment to “best practice” treatment strategies, millions of people die each year from various forms of cancer and predicted incidence trends continue to climb, suggesting more effective cancer management approaches are required. In response to this critical need for improved cancer therapeutics, there has been increased attention towards the use of precision medicine to limit off-target effects and increase patient quality of life during treatment. The mainstay of treating most cancers is often surgical resection, radiation or chemotherapy, along with newer approaches such as immunotherapy (Baskar et al., 2012; Yildizhan et al., 2018). Due to the invasive nature and broad spectrum target of these common therapies, they are all prone to off-target toxicity to surrounding healthy tissue, ultimately resulting in unwanted and detrimental side-effects for the patient. More recent advances in cancer treatment include immunotherapy approaches, which help the patients’ immune system recognize tumor cells for destruction, and chemotherapy-coupled approaches, which either sensitise tumor cells to chemotherapeutic drugs or allow direct targeting of chemotherapeutic agents to the interior of the tumor cell (Sekhon et al., 2017; Pereira et al., 2018). These types of therapies have been shown to exert highly specific tumor cell killing with minimal effect on adjacent healthy normal cells. In the case of approaches which utilize tumor-specific targeting, this is often attributed to the reduced drug concentrations required due to more efficient intracellular delivery to tumor cells as the targeting agents generally trigger internalization after binding (Zhu et al., 2015; Catuogno et al., 2016). Even with the advancement towards more targeted therapy that these approaches represent, chemotherapy-coupled therapeutics can still have toxic effects on healthy tissue due to off-target actions of the drugs themselves or through the build-up of potentially harmful nanoparticles utilized for delivery of the drug into the cell (Hossain et al., 2011; Patra et al., 2018). It is therefore urgent that targeted therapeutics, that not only halt or reverse tumorigenesis, but also do not require coupling to chemotherapeutic drugs or nanoparticles, are developed to treat aggressive cancers with high specificity.

To this end, highly specific molecular targeting agents are of great interest to treat cancer by targeting intracellular signalling pathways directly, either through receptor binding leading to activation/inhibition, or *via* internalization and targeting to specific subcellular compartments. DNA aptamers represent a promising therapeutic avenue in this arena that can be used for both specific cell surface targeting, and in some cases may elicit a functional intracellular response upon binding and internalization *via* target molecules on the surface of tumor cells, ultimately resulting in tumor cell death (Kim et al., 2018; Pang et al., 2018). This review will specifically examine the latter, focusing on the intracellular functions of internalized DNA aptamers in tumor cells. Many aptamers that are not internalized do have downstream cytotoxic activity, through receptor binding and activation/inhibition of cellular pathways, however these effects are mediated through signalling blockades and not *via* intrinsic intracellular mechanisms (Talbot et al., 2011) and are not the intended focus of this discussion as they are reviewed extensively elsewhere (Dua and Kim, 2011; Mercier et al., 2017).

## Aptamers Are Highly Selective Molecular Targeting Agents

First developed in 1990, aptamers are short single-stranded stretches of nucleic acids, usually ranging between 20–80 nucleotides, that can selectively bind to a specific target, due to their ability to form unique hairpin-like three-dimensional structures. Aptamers can be designed against many targets including proteins, carbohydrates, small molecules, peptide toxins, and even whole cells (Yang et al., 2011; McKeague and Derosa, 2012; Tabarzad and Jafari, 2016). Aptamers have been developed to recognize and treat several diseases, and to date they recognize a diverse range of targets applicable to therapeutic intervention including proteins, whole cells, tissue types, bacteria, and viruses. Aptamers can be generated from either DNA or RNA, and more recently peptide aptamers have been developed, however these consist of short amino acid stretches embedded in a protein scaffold (Mascini et al., 2012) and are thus largely a different entity. Although RNA aptamers function in a very similar fashion to DNA aptamers, they are developed using slightly different methods and often require chemical modification for *in vivo* stability, whereas many DNA aptamers may not (Zhu et al., 2015). This review will largely concentrate on functional DNA aptamers.

### SELEX (Systematic Evolution of Ligands by Exponential Enrichment)

**Figure 1:**
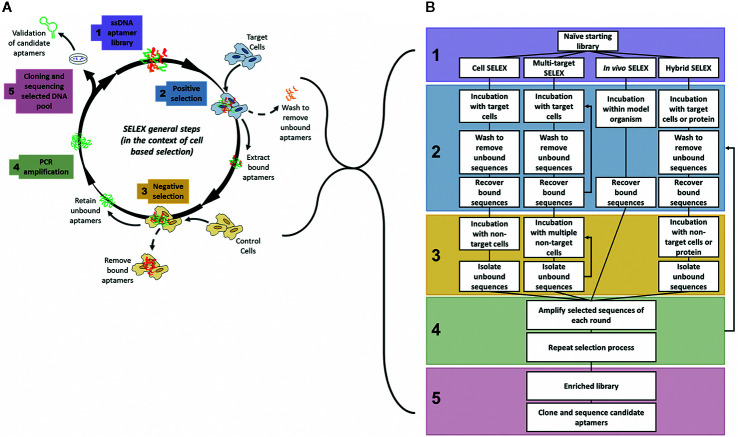
General steps of aptamer selection. **(A)** SELEX follows 5 main steps to generate a highly specific targeting aptamer. Step 1 involves the synthesis of a naïve library, with on average 10^18^ random aptamer sequences per library. The 2^nd^ step is where selection begins, often with a positive round where the naïve library is targeted to cells or proteins of interest, after an incubation period with the target the bound aptamer sequences are recovered from the target cells. The 3^rd^ main step of SELEX is the negative selection step where the positively selected pool of sequences is incubated with non-target cells or proteins to separate out off-target aptamer sequences. Only unbound aptamers are kept from this step. The 4^th^ step is to amplify the selected pool and repeat the cycle of positive and negative selection rounds until the aptamer pool is sufficiently enriched to only contain sequences that bind specifically to the target. In the 5^th^ and final step of SELEX the enriched pool is cloned and sequenced to identify candidate aptamers (Ellington and Szostak, 1990; Tuerk and Gold, 1990; Shangguan et al., 2006). **(B)** Comparison of various types of SELEX, grouped by main steps of generation. Note: In multi-target SELEX either or both of the selection steps may be repeated through multiple targets, either sequentially or as a combined pool.

Aptamers are generated by an iterative process known as SELEX (Systematic Evolution of Ligands by Exponential Enrichment), which is a process to iteratively derive an aptamer that is highly specific for a target from a pool of unselected aptamers using successive positive and negative selection steps (**Figure 1**). First developed in 1990 (Ellington and Szostak, 1990; Tuerk and Gold, 1990), the SELEX process has undergone various evolutions to optimise and enhance both the binding ability and target specificity of the developed aptamers. Therefore, depending on the target and intended use of the aptamer, the specific details of the generation protocol can vary. For example, selection processes may require more stringent washing steps, such as washing with highly concentrated salt solutions to remove weakly bound aptamers (Thiel et al., 2015) or washing with enzymatic solutions to digest cell surface proteins and externally bound aptamers (Iaboni et al., 2016), in order to isolate only aptamers that have been internalized inside target cells, or may involve multiple positive selection rounds utilizing both target cells and purified target proteins to ensure that the resultant aptamer binds only to a specific protein of interest (Hicke et al., 2001; Zhu et al., 2017). Despite this, all of the methods have some common features and in particular they all follow an iterative process involving successive rounds of positive and negative selection to develop target specificity. In general, a large library of random ssDNA (or RNA) sequences (aptamers) are incubated with the intended target, to which the library binds through structural recognition based on the aptamers’ inherent 3D conformation (**Figure 1**). The bound sequences are then recovered and amplified by PCR. This selection is refined by multiple subsequent positive and negative selection rounds, where aptamers from the initial library are incubated first with the target sample (often an immobilized protein of interest), retaining any bound sequences (positive selection). This bound pool is then incubated with a non-target sample (a structurally similar protein or mutant variant of the target) and any bound sequences are removed (negative selection), keeping only the unbound fraction of the aptamer pool. In some protocols, negative selection may precede positive selection to reduce the quantity of possible aptamer targets available for positive selection. The aptamer pool is then amplified by PCR and the selection rounds are repeated between 10–20 times. The number of rounds is dependent upon the nature of the target. SELEX protocols generally include a selection monitoring step to track the evolution process and SELEX rounds are usually repeated until the aptamer pool is enriched to such a level that the amount of target binding plateaus, leaving a final selected pool of aptamers highly specific to the intended target. This iterative positive and negative selection process is what endows aptamers with their highly specific targeting ability (Komarova and Kuznetsov, 2019). These aptamer sequences are then amplified by PCR, subcloned, and subjected to high throughput sequencing to identify and create a purified aptamer library specific for a certain marker. The two main classes of SELEX used to generate aptamers are protein-SELEX or cell-SELEX.

#### Protein-SELEX

Protein-targeted SELEX generates highly specific aptamers targeting an already identified biomarker. Protein-SELEX mostly requires only appropriate immobilization of the target protein (recombinantly expressed or purified from a target sample) during selection rounds for effective enrichment by SELEX. For cancer therapy, aptamers derived *via* protein-SELEX can create issues relating to target recognition *in vivo* due to conformational changes or modifications not maintained during the *in vitro* selection process (Chen et al., 2016), and furthermore target proteins may be abundant on the surface of many types of cells/cancers and therefore extensive prior knowledge of the target, such as differential isoform expression or post-translational modifications, is needed to generate truly cancer-specific aptamers *via* this technique.

#### Cell-SELEX

To overcome many of these issues, the SELEX process can also be performed in a modified context, known as whole cell-SELEX (Shangguan et al., 2006) (**Figure 1A**), where an aptamer library is incubated not with a purified protein, but with a certain cell type (such as a tumor cell), and then negatively selected using another cell type (such as a normal non-cancerous cell) to generate aptamers specific for the tumor cells (Cerchia and de Franciscis, 2010). This method facilitates the development of specific probes that distinguish between the molecular signatures of normal and tumor cells, and may be used for diagnosis, delivery of treatments, or to identify novel tumor biomarkers. Cell-SELEX methods may also be more appropriate to generate highly specific cancer targeting agents that recognize internalizing receptors without prior knowledge of overexpressed surface targets/biomarkers. The various types of SELEX discussed throughout this review are summarized in **Figure 1B**.

### Aptamers Have Many Advantageous Properties for Use as Therapeutic Agents

The use of aptamers as therapeutic agents has many advantages compared to other conventional biomolecules already used for various applications of research, i.e. monoclonal antibodies or cytotoxic drugs. These include ease of production and scale up by chemical synthesis, limited batch-to-batch variation, high-specificity for target molecules, long-term stability in a range of temperatures and solvents, simplicity of conjugation to other molecules of interest, and relatively low immunogenicity (Kong and Byun, 2013; Röthlisberger and Hollenstein, 2018).

Due to their similar target specificity and ability to bind to biomarkers through structural recognition in a similar fashion to conventional antibodies, aptamers are often described as “chemical antibodies”. In addition, the binding specificity and strength of interaction between aptamers and their molecular targets is sometimes even stronger than that observed between antibodies and their targets (Jenison et al., 1994). As such, the most similar therapeutic agent used in current clinical practice that can be compared to aptamers are monoclonal antibodies (mAbs), such as trastuzumab (Herceptin)—the first FDA approved monoclonal antibody-based treatment for solid tumors (Dillman, 1999), which targets HER2 and blocks downstream signalling resulting in reduced cell growth and cytotoxicity (Molina et al., 2001). Other developed mAbs are widely used in current clinical practice for treating various types of cancer, but often have negative side effects related to their use (**Table 1** compares the useful therapeutic features of aptamers and mAbs).

**Table 1:** Characteristics required for tumor targeting agents; comparison of nucleic acid aptamers and antibody-based therapeutics.

	Aptamers	Antibodies
**Average size**	< 30 kDa	~ 150 kDa
**Targeting specificity**	Wide range of possible targets. Can distinguish between protein isoforms or isogenic cell types.	Immunogenic based, selectivity can be altered based on host animal.
**Binding strength**	Similar to antibodies depending on target and generation method. Kd often in nanomolar range.	Similar to aptamers depending on target epitope and mono or polyclonality. Kd often in nanomolar range
**Generation method/time**	Generated using chemically synthesized nucleic acid libraries	Generated in animal hosts (*in vivo*) requiring purification
**Batch-to-batch variability**	No variation due to precise chemical synthesis	Batch-to-batch variation is commonly observed
**Nuclease resistance**	Non-resistant, can be modified to resist degradation	Resistant to nuclease degradation
**Common off-target effects**	Chemical modifications can be toxic if accumulated in tissue	Immune reaction, low membrane permeability/internalization ability
**References**	(Ali et al., 2019)	(Keefe et al., 2010; Banerjee and Nilsen-Hamilton, 2013)

Unlike antibodies which can take months to generate, aptamers are relatively fast and easy to produce, although the initial generation can be a time-consuming process, there is no requirement for animal hosts to make an aptamer compared to antibodies and the final product can be synthesized directly, leading to ease of production scale up and little to no batch-to-batch variation. Another advantage aptamers have over mAbs is that they have low or no immunogenicity (Sun et al., 2014). This is in part due to their small size, which minimizes potential toxicity that might be caused by excess aptamers or their conjugates. On the other hand, the small size (averages between 5–15 kDa) of aptamers may also be a disadvantage for *in vivo* use, where renal filtration may occur before the aptamer can reach its intended target site (Catuogno and Esposito, 2017; Wu et al., 2017). In contrast, conventional mAbs are much larger in size compared to aptamers, resulting in longer circulation times *in vivo*, and reduced internalization capability. Conjugation of aptamers to larger molecules (most commonly polyethylene glycol—PEG) can however extend the circulation time of aptamers *in vivo*, although this increased ability to remain within the circulatory system may unfortunately ultimately be a disadvantage, as aptamers, especially those made of RNA, are susceptible to nuclease degradation. Chemically modified aptamers are however relatively easy to produce and can reduce vulnerability to nucleases *in vivo* (Wu et al., 2017).

Finally, in comparison to the retarded cellular internalization of large mAbs, aptamers are often readily taken up by cells, through a number of mechanisms, most commonly endocytosis and micropinocytosis (Yoon and Rossi, 2018). Aptamer uptake is ultimately determined by the specific interaction with their target and are therefore very appealing for intracellular targeting applications such as tumor specific cell internalization, molecular pathway inhibition, or induction of apoptosis. In all, most of the advantages of aptamers directly support their development as therapeutic agents and most of the disadvantages are easily overcome in the manufacture process by chemical conjugation or modification (relevant modifications are summarized in **Table 2**). It is important to note however that chemical modifications of aptamers can have deleterious effects upon the proposed safety and efficacy of aptamers as therapeutics, with issues such as increased renal accumulation and non-degradation arising when aptamers are conjugated to other molecules (Wang et al., 2011a). But these costs can be weighed up with therapeutic efficacy when aptamers reach clinical trial stages.

**Table 2:** Common chemical modifications for improvement of aptamer performance in pharmacological studies.

Aptamer modification	Desired effect	Off-target effects
**PEGylation**	Longer circulation time	Allergic reactions
**Locked nucleic acids**	Nuclease resistance	Hepatotoxicity
**2’ sugar ring substitution**	Nuclease resistance	May activate pattern recognition receptors
**Phosphorothioate replacement**	Improved binding & thermal stability	Activation of the complement system
**Spiegelmers**	Improved binding & nuclease resistance	None reported
**Slow off-rate-modified aptamers**	Improved binding & nuclease resistance	None reported
**Conjugation to nanomaterials**	Improved functionality & stability	Nanomaterial-mediated toxicity
**References**	(Sun et al., 2014; Odeh et al., 2019)	(Ruckman et al., 1998; Ni et al., 2017)

## Aptamer Therapeutics in Current Cancer Research—Clinical Use of “Non-Functional” Aptamers

Initially aptamers were generated as simple targeting or drug delivery agents, envisioned for use as diagnostic tools, in which the highly specific target recognition of aptamers could be used for example to characterize cell/tissue types for real-time biomarker analysis or for detection of infectious microorganisms or viruses, or as targeting agents able to deliver a cytotoxic payload to a specific cell type of interest. For example, aptamers have often been co-opted for target visualization by using fluorescently labelled aptamers, which can show localization or abundance of the target upon aptamer binding and subsequent fluorescence microscopy (Mittal et al., 2017; Hori et al., 2018; Yoon and Rossi, 2018). These have been useful in cancer diagnostic studies where tumor cells are specifically identified by fluorescently-labelled aptamers binding to their respective tumor-specific targets (Benedetto et al., 2015; Chandrasekaran et al., 2016) and may be particularly amenable to high-throughput automated imaging platforms such as those used currently for diagnostics [eg. autoPap for automated pap-smear diagnostics (Bengtsson and Malm, 2014)]. Fluorescent aptamers can also be used for monitoring cellular internalization and subcellular translocation of target proteins, as bound aptamers move to specific organelles based upon the movement of the target within the cell (Gomes de Castro et al., 2017). These can be detected by methods that allow visualization of conjugated fluorophores or nanoparticles such as fluorescence microscopy, magnetic resonance imaging, or ultrasound imaging.

**Figure 2:**
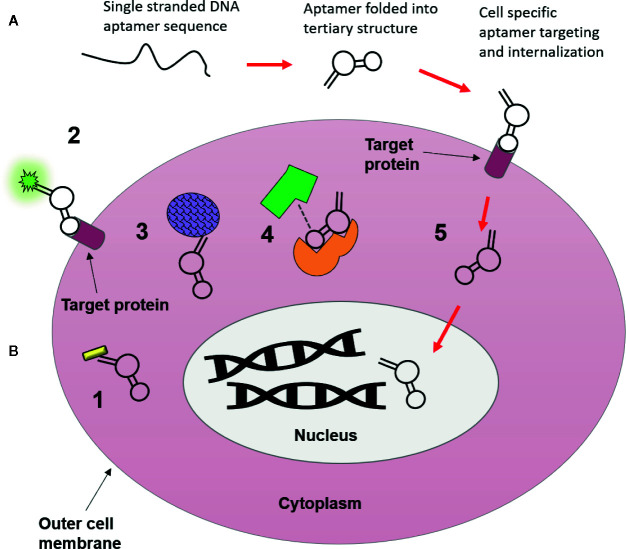
Possible uses of nucleic acid aptamers as targeting agents. **(A)** Aptamers begin as single strands of nucleic acids, which then fold into a tertiary structure that is highly specific for molecular targets they are generated towards. Following their binding they can be used for intracellular targeting and as novel therapeutics visualized in B. **(B)** 1. Conjugation to nanomaterials (gold nanorod) for drug free therapy (photothermally induced cell death) upon internalization (Chandrasekaran et al., 2016). 2. Target localization *via* aptamer conjugated fluorophores (Bouvier-Müller and Ducongé, 2018). 3. Conjugation to nanomaterials (drug delivery vehicle i.e. silicone or lipid nanoparticles) to deliver chemotherapeutic payloads to cytoplasm of tumor cells, resulting in fewer off-target side effects (Zhao et al., 2017). 4. Molecular target inhibition, aptamers may block or activate molecular pathways through binding interactions with target proteins, where binding of aptamers may inhibit target-ligand binding (Lee et al., 2005). 5. Cellular internalization and subcellular translocation, aptamers are capable of moving to specific organelles (i.e. nucleus) based upon movement of their bound target within the cell (Aptekar et al., 2015).

Progress in aptamer generation techniques and characterization methods in the last few years has since given rise to more and more “functional” aptamers, capable of directly activating or inhibiting molecular targets for downstream effects. In this review a functional aptamer is defined as an aptamer that binds to and is internalized into tumor cells, and further activates cytostatic signalling pathways *via* intracellular targets. Despite their subsequent definition as “non-functional” aptamers, many of the aptamers that fall outside this definition because they do not internalize or they evoke an anti-tumor response through receptor binding on the cell surface alone, have a range of potential applications as theranostic agents (summarized graphically in **Figure 2**), which will be discussed in the following sections.

### Aptamers That Function Extracellularly, Through Specific Target Antagonism

The molecular recognition of aptamers for their target is so specific that some aptamers are capable of precisely distinguishing between two protein isoforms, which can be exploited therapeutically if one isoform is related to disease and the other is not. This is exemplified by the FDA approved therapeutic aptamer Macugen, which recognizes the predominantly expressed isoform of vascular endothelial growth factor (VEGF), the pro-angiogenic VEGF165a. Macugen shows very little affinity for smaller VEGF isoforms lacking the heparin binding domains or the alternatively spliced anti-angiogenic VEGF165b isoform (Lee et al., 2005; Ng et al., 2006; Amadio et al., 2016). In this way Macugen binds specifically to the pro-angiogenic isoform of VEGF when administered by intravitreal injection, acting as a VEGF antagonist, thereby resulting in reduced blood vessel growth and permeability/leakage, both of which are hallmarks of neovascular age-related macular degeneration.

In similar fashion, other aptamers have been shown to exhibit competitive binding with their targets, resulting in the antagonism or activation of molecular pathways with therapeutic potential. For example, the CL4 aptamer inhibits the growth of triple negative breast cancer (TNBC) cells by impairing binding of epidermal growth factor receptor (EGFR) to integrin αvβ3 (Camorani et al., 2017). CL4 binding on the cell membrane resulted in antagonism of vascularization and cell adhesion pathways normally regulated by the binding of EGFR to integrin αvβ3, resulting in reduced tumor growth. The mechanisms of action and further implications of these two aptamers in the arena of developing functional therapeutic aptamers will be discussed further in later sections, but they stand as excellent examples of the ability of aptamers to elicit a functional therapeutic response even in an extracellular context.

### Aptamer Conjugation to Active Molecules

Due to their highly specific recognition ability, aptamers are able to act as targeting agents for a range of biologically active payloads. In particular, they have been demonstrated to be useful in pre-treatment tumor sensitization, in which chimeric aptamers conjugated to siRNA molecules facilitate targeted siRNA delivery to the interior of the cell, prior to drug-delivery (Dassie et al., 2009) or for direct drug-delivery through conjugation to nanoparticles containing chemotherapeutic payloads (Hu et al., 2012; Zhao et al., 2017).

#### Aptamer Conjugation to Nanomaterials

Non-functional aptamers which do not inherently elicit a functional response in cells can still be utilized as highly efficient targeting agents through conjugation to nanomaterials. In this way, non-functional aptamers can be rendered “functional”, enabling cell specific drug delivery or eliciting cell death. Examples of these technologies include conjugation to nanoparticles (e.g. gold nanorods) for photothermal-induced cell death upon internalization and laser activation (Chandrasekaran et al., 2016) or conjugation to nanoparticles filled with chemotherapeutic payloads (i.e. silicone/lipid nanoparticles) to deliver drugs directly to the cytoplasm of tumor cells (Zhao et al., 2017), resulting in fewer off-target side effects. For example, aptamers targeting the cell surface of TNBC cells were conjugated directly to gold-nanorods which resonate in response to specific near infra-red wavelengths. Activation of these particles resulted in localized heat production and photothermal-induced cell death specifically in the tumor cells and with no death observed in isogenic normal cells (Chandrasekaran et al., 2016). Similarly, the aptamers S2.2 and AS1411 (targeted against cell surface MUC1 and nucleolin proteins, respectively) were dually conjugated to thermosensitive liposome nanostructures, encapsulating ammonium bicarbonate, and docetaxel, coated in gold nanoshells. Upon binding and internalization into MCF-7 breast cancer cells *via* aptamer-target interaction and following laser activation these nanoparticles released carbon dioxide bubbles within the cell, which were able to be visualized by ultrasound imaging for tumor localization/diagnosis, while concurrently releasing their chemotherapeutic payloads into the cells in which they had been internalized (Zhao et al., 2017), resulting in efficient tumor-specific cell death.

These types of approaches which render “non-functional” aptamers functional are extensively reviewed elsewhere (Wang et al., 2011b; Catuogno and Esposito, 2017; Hori et al., 2018), so the remaining sections will focus upon aptamers that interact with the cell and its intracellular components directly to eliminate or halt cancerous cell growth.

## Highly Specific and Efficiently Internalized Cancer-Targeting Aptamers

To generate functional aptamers as efficient cancer therapeutics several criteria must be met. First, the aptamer must efficiently bind to and then internalize into cells to allow the aptamer to act upon intracellular targets and pathways. This requires appropriate cancer specific cell surface target expression, and effective stability to ensure that the aptamer is not immediately degraded prior to and upon internalization into tumor cells. The first part of this, specificity, can be largely controlled by appropriate and careful negative selection rounds during the SELEX process, such as the use of isogenic cells (see below) (Chandrasekaran et al., 2016) but also by the particular method of SELEX chosen (see **Figure 1B**).

### Variations of Cell-SELEX Generate Functional Aptamers With Unique Properties

#### Whole Cell-SELEX

Most aptamers generated towards tumor cells or biomarkers are developed using adaptations of whole cell-SELEX, which allows for selection of target molecules without prior knowledge of a specific target on the cell lines used. Aptamers developed using cell-SELEX are often targeted to cell surface or secreted proteins and are therefore often efficiently internalized into target cells by endocytosis following receptor binding. Examples of this include the TNBC specific aptamer KW16-13 (Chandrasekaran et al., 2016), the epithelial ovarian cancer specific aptamers RLA01, RLA02, and RLA03 (Benedetto et al., 2015); and the HER-2 positive breast cancer specific aptamer MF3 (Liu et al., 2018).

These aptamers exhibit exquisite target specificity—where aptamer binding was examined by flow cytometry and/or fluorescence microscopy, each distinguishing between normal, pre-malignant or metastatic cell lines of their relevant target cancer type and in some cases distinguishing between other types of cancer (Benedetto et al., 2015). In the case of KW16-13 this specificity (~9-fold more tumor specificity during selection and ~74-fold more cell death when conjugated to photothermal-GNRs, compared to that of isogenic normal cells) was achieved through the use of isogenic cell lines during the cell-SELEX process (Chandrasekaran et al., 2016). In this case the isogenic cells were cells in which the target tumor line originated from the same genetic background as the normal negative selection line.

Another aptamer that powerfully distinguishes between normal, non-metastatic and metastatic prostate cancer cell lines is DML-7, which was generated to target the metastatic prostate cancer cell line DU145 using cell-SELEX (Duan et al., 2016). DML-7 was shown to bind to patient tissue samples and was more closely associated with increased metastatic potential, with minimal binding to non-metastatic and normal pancreatic cancer tissue samples observed. This suggests DML-7 may have feasibility as a direct diagnostic tool. Additionally, DML-7 was shown to exhibit efficient internalization into metastatic cell lines *via* receptor-mediated endocytosis (Duan et al., 2016), making it a strong candidate for aptamer-mediated drug delivery applications in metastatic prostate cancer cells. DML-7 epitomizes the most straightforward therapeutic applications of cell internalizing aptamers and demonstrates clearly the strong target specificity aptamers can offer. Unfortunately, it does not appear to exert any biological function upon target cells following its internalization and is therefore best suited for diagnostic and drug-delivery applications.

One drawback of each of these aptamers is that since they were derived using cell-SELEX, the target molecule of the final aptamer remains unknown until it can be confirmed by other means, often by pulldown and subsequent mass spectrometric protein identification. These techniques can yield many candidate target hits of varying affinities (Van Simaeys et al., 2014; Yoon et al., 2017) that require subsequent confirmation through direct binding studies. In spite of this target ambiguity, these aptamers do represent a new highly specific class of tumor targeting agents that have potential to be used in diagnostic and intracellular therapeutic applications, particularly for aptamers which appear to be efficiently internalized in the target cell of choice.

#### Multi-Target SELEX

As alluded to above, aptamer specificity is largely generated during the initial selection process and the SELEX approach is continuously being iteratively enhanced for this purpose. One approach to generate aptamers that display high cancer type specificity is multi-target cell SELEX, which uses several distinct cell lines in sequential selection steps or a pooled target negative selection step (Hung et al., 2014; Zou et al., 2018). For example, an automated on-chip cell-SELEX based approach to develop an ovarian cancer specific aptamer utilized four different ovarian cancer cell lines, where one line was chosen for positive selection, and subsequent selection steps utilized the remaining three ovarian cancer cell lines for negative selection in each SELEX round (Hung et al., 2014). The result of this process should ensure that the selected aptamers recognize distinct ovarian cancer subtypes while disregarding other ovarian cancer cell types. The resulting 13 aptamers of this novel SELEX procedure demonstrated high ovarian cancer cell targeting specificity with low dissociation constants and highly specific target cell capture rates. The aptamers generated in this study were not characterized beyond binding affinity and cell selectivity, but due to their target specific cell binding and internalization, they may be of interest in future studies for elucidation of ovarian cancer biomarkers, for ovarian cancer specific intracellular targeting or for targeted drug delivery. These aptamers also show great potential for development of an ovarian cancer screening tool, due to their capability to capture target cells in 30 min when bound to magnetic beads (Hung et al., 2014).

A similar pooled multi-target whole-cell SELEX approach was used to generate aptamers specific to different stages of E. Coli O157:H7, an important food borne pathogen related to public health (Zou et al., 2018). The approach used 14 selection rounds and included three counter-selection rounds to generate aptamers with high affinity and specificity to different phases of O157:H7 growth. Although described in bacterial cells, the pooled SELEX method is clearly exemplified in this approach in which the method is used to increase selection of high affinity aptamers by increasing selection pressure through the use of a highly complex sample composition. This coupled with the large number of aptamer sequences in the initial library, ensured that selection rounds enrich the most highly bound and specific aptamers from the pool rather than enriching the most abundant or easy to replicate (Zou et al., 2018). Therefore, this novel SELEX approach exemplifies the advantages of continually enhancing SELEX approaches in the pursuit of generating highly specific cancer targeting aptamers and subsequent development of novel diagnostic tools.

#### *In Vivo* SELEX

When aptamers are generated, *in vivo* stability and targeting specificity are usually the most difficult functions to actively select for using standard SELEX techniques. *In vivo* SELEX was developed to generate robustly efficient and specific DNA aptamers *in vivo* (Chen et al., 2019). In this technique, nude mice bearing luciferase labelled PC3 bone metastases, were injected with a random aptamer library which was allowed to circulate for 4 h. Only luciferase-labelled bone marrow metastases were collected from the sacrificed mice and cell internalized aptamers were extracted, which led to the generation of a highly selective *in vivo* intracellular targeting aptamer, designated “PB” (Chen et al., 2019). This novel generation method selected for several advantageous characteristics that were apparent in PB; selected aptamers inherently had appropriate serum stability to find and target bone metastases, while off-target aptamers within the pool were negatively selected against by only isolating aptamer sequences bound and internalized into luciferase labelled PC3 cells. PB was shown to be internalized by endocytosis but unlike most internalized aptamers PB managed to avoid lysosomal degradation, escaping after 6 h, making it an excellent targeting agent for therapeutic delivery and subsequent intracellular targeting. While this method of SELEX is arguably more time consuming and difficult to carry out, it overcomes a large hurdle in *in vitro* aptamer targeting where aptamers generated to cell lines or purified proteins tend to accumulate in other organs and may exhibit off-target side effects.

### Intracellular Release and Lysosomal Escape Are Critical Properties of Functional Aptamers

After internalization, the second important requirement of a functional aptamer depending on the route of uptake, is an ability to escape endosomal degradation and release into the cytoplasm to target intracellular molecules. This function was observed in *in vivo* SELEX generated aptmer PB, which escaped lysomal degradation after 6 h and is most prominently described as a function of aptamer AS1411, which binds to nucleolin on the cell surface and has been shown to reduce cell proliferation though induction of apoptosis (Wu et al., 2017). This function for the most part is achieved by utilizing an appropriate target. As mentioned, the best example of this is AS1411, which is known to efficiently escape into the cytoplasm, followed by translocation to the nucleus (Bates et al., 2009; Reyes-Reyes et al., 2015; Wu et al., 2017), however the mechanisms underpinning this remains to be fully elucidated. Studies suggest there may be more than one mechanism of entry depending on the cell type, with both micropinocytosis coupled with leaky macropinosomes observed in DU145 prostate cancer cells and non-micropinocytosis-mediated mechanisms in Hs27 non-malignant skin fibroblasts. If the mechanisms of AS1411 effective intracellular aptamer release can be appropriately determined they may point to an efficient mechanism which can be exploited to avoid lysosomal degradation after endocytosis-mediated internalization in the development of future therapeutic aptamers.

An excellent example of efficient aptamer internalization and subsequent functional anti-cancer activity uses an aptamer complex developed for highly efficient internalization into breast and prostate cancers, based upon common receptor overexpression (Abnous et al., 2017). This aptamer complex is composed of three therapeutic aptamers, each known to bind to overexpressed molecular targets on cancer cells, aptamers AS1411 – targeting nucleolin, IDA – targeting the integrin α6β4 receptor, and apMNK2F – which selectively inhibits MAP kinase interacting kinase 1. Internalization of the aptamer nanocomplex was facilitated by both AS1411 and IDA and resulted in widespread cytoplasmic localization, demonstrating the critical ability to escape endosomal degradation, followed by nucleolin-mediated specific delivery to the nucleus of target cells. Consistent with each of these aptamers having been previously shown to exert anti-cancer action upon binding/internalization, the nanocomplex alone was able to effectively suppress the growth of tumors *in vivo*. This complex, while exemplifying the ability for aptamers to internalize into cells and target intracellular organelles and molecules, also reinforces the idea that aptamers can treat cancer in a cell specific manner while minimising toxic off-target effects to healthy surrounding tissue usually associated with the use of cytotoxic agents.

### Aptamers Selected Towards Specific Target Biomarkers

As discussed previously, cell-based SELEX methods do not require previous knowledge of cell specific targets or biomarkers, which, while advantageous for the selection process often makes it challenging to identify the aptamer’s molecular targets. In turn, determining functional ability without target information can often be problematic. A particular advantage to identifying the targets of aptamers generated by cell-SELEX is that once the specific molecular target of the aptamer is known, aptamers can be used to identify the distinct molecular mechanisms at play within tumor cells, and further research can be carried out to develop therapeutics that target these mechanisms specifically to exert anti-cancer activity. This of course is limited to the ability of the target molecule to control oncogenic function, but nevertheless the aptamer must be able to act as a ligand, inhibitor, or regulator of its target by binding at sites necessary to protein function or inhibiting oncogenic complex formation. Therein, the final requirement of functional therapeutic aptamers is their ability to elicit anti-cancer activity within target cells.

One excellent example of target identification comes from the aptamer CL4, which was generated by cell-SELEX (Esposito et al., 2011; Camorani et al., 2017) and was subsequently shown to bind to EGFR and inhibit formation of EFGR-integrin αvβ3 complexes. CL4 bound to EGFR expressed on the cell surface of various TNBC cell lines and inhibited EFGR-integrin αvβ3 complex formation, which in turn inhibited matrix associated vessel formation and cell adhesion in 3D culture models. This ability to inhibit vasculogenic mimicry ability and ultimately tumor growth was also shown in xenograft mouse models (Camorani et al., 2017). Inhibition of tumor growth was only seen in CL4 treated mice, with no apparent off-target toxicity shown by either scrambled or target aptamers. Additionally, CL4 exhibited better tumor inhibition than the cytotoxic agents erlotinib (a tyronsine kinase inhibitor) and cetuximab (a monoclonal antibody) (Camorani et al., 2017), both FDA approved, further cementing this biotherapeutic aptamer as a promising therapeutic alternative.

While CL4 was not initially generated to specifically target EGFR, subsequent identification of the target led to further study to characterize the functional anti-cancer action of this aptamer in EGFR overexpressing tumors, supporting the idea that deeper understanding of molecular targets and interactors can lead to more specific therapeutic investigation of cell internalizing aptamers to uncover the intracellular signalling mechanisms that drive tumor growth, metastasis or survival.

#### Hybrid-SELEX

In addition to aptamers generated by cell-SELEX several other protocols are currently used in research including traditional protein-targeting SELEX (Tuerk and Gold, 1990) and more recently a combination of cell and protein targeting called hybrid-SELEX (**Figure 1B**) (Hicke et al., 2001; Mercier et al., 2017). Aptamers generated towards molecular targets using purified proteins tend to have trouble targeting native or active forms of the biomarker in cells due to availability, conformational and post-translational variations, and subcellular localization issues. Hybrid-SELEX aims to overcome these challenges, while maintaining specific molecular target selection (Mallikaratchy, 2017; Zhu et al., 2017).

One example of hybrid-SELEX that overcomes the aforementioned disadvantage of protein targeted SELEX, incorporates cell specific targeting and non-specific negative cell targeting rounds followed by further selection rounds using purified proteins of the intended target. An example of this version of hybrid-SELEX was utilized to generate the aptamers HL-1 and HL-2 to target the Maver-1 B-cell lymphoma line and negatively selected against the Jeko-1 mantle cell lymphoma line, followed by subsequent positive selection with immunoglobulin lambda-like polypeptide 5—a gold standard biomarker for B cell lymphoma diagnosis (Li et al., 2017). This ensured that the generated aptamers were not only target specific, but also extremely precisely targeted to minimize binding to normal B cells to reduce possible adverse effects to healthy cells. These aptamers were not only cancer subtype specific but also selected to be tumor cell-specific based on known biomarker presentation, and upon internalization had anti-proliferative effect. Excitingly one of these aptamers, HL-1, was shown to exert anti-cancer activity upon binding, and results showed that internalization of the aptamer into Maver-1 cells induced cell cycle arrest, resulting in tumor growth inhibition (Li et al., 2017). Investigation of the mechanism of HL-1 internalization into cells showed that the aptamer was unable to elicit cytostatic activity when endocytosis was blocked by an inhibitor, indicating that endocytosis-mediated internalization was essential for the anti-cancer action of HL-1.

Further refinement of protein-targeted SELEX has also been used to overcome the trouble that protein-SELEX derived aptamers have in targeting native or active forms of the biomarker in cells. This approach takes the idea of hybrid-SELEX but utilizes stably mutated active forms of the protein rather than cells during selection and has been shown to be effective in generating aptamers highly specific for proteins both in native and non-native forms. Examples of aptamers generated by this modified version of protein-targeting SELEX are the aptamers WT15 and SM20 (Blake et al., 2009), which were generated to target the plasminogen activator inhibitor-1 (PAI-1) wild type protein, or a stably mutated version of PAI-1 which maintains the protein in its active conformation, respectively. Utilizing two separate selections against wild type PAI-1 and the stably mutated version of PAI-1, the aptamers generated from both selections were shown to be highly specific for the wild type version of the protein. This application of a differential protein-targeting hybrid-SELEX technique can thereby overcome the common limitation of protein-based SELEX, enabling targeting to proteins in conformations that are more likely to be expressed *in vivo*, without the need for adding cells into the selection rounds—reducing some time and cost associated with performing cell-SELEX. Further characterization of WT15 and SM20 demonstrated that both aptamers were able to competitively bind to PAI-1, subsequently inhibiting its binding to heparin and vitronectin. Transfection of these aptamers into breast and endothelial cancer cells exhibited therapeutic potential, resulting in reduced cellular migration, invasion, and angiogenesis upon functional aptamer intracellular targeting (Fortenberry et al., 2016). Although these aptamers were not shown to efficiently internalize into cells, transfection directly into cells showed significant benefit as a therapeutic agent in contrast to traditional chemotherapeutic drugs.

Target mediated aptamer generation has advanced significantly both through the use of methods that actively select for native protein targets, and through adoption of methods that also involve a cellular targeting round. These examples show that aptamers are now rarely made without regard for future application, demonstrating a shift in attitude away from largely diagnostic applications towards using aptamers themselves as novel therapeutics (Zhou and Rossi, 2017).

## Applications of Aptamers in Precision Medicine Therapy for Cancer—Aptamers in Clinical Trials

It is well known that aptamers can differentiate between protein isoforms, some studies show aptamer binding can also be affected due to protein modification and available binding sites (Deng et al., 2014), demonstrating the selective targeting ability of aptamers as intracellular targeting agents for alternative cancer therapeutics. Aptamers have already been developed for many common cancer targets and can act as molecular switches, or may enable intracellular targeting to facilitate drug delivery, or support precision therapy approaches through targeted molecular recognition (Sun et al., 2014). Aptamers that efficiently bind and target intracellular pathways are of particular therapeutic interest, as this may uncover cancer specific mechanisms that can be hijacked for therapeutic benefit. Although not many have yet made it to clinical trials or achieved FDA approval, there is certainly potential to use aptamers more widely in novel therapeutic approaches with conjugation to anti-cancer drug-carriers and nanoparticles, and potentially on their own as molecular antagonists with therapeutic benefit. The most prominent examples of cancer targeting aptamers from clinical trials are detailed below and summarized in **Table 3**.

**Table 3:** Comparison of prolific aptamers commonly advertised as promising therapeutic agents for cancer.

	AS1411	NOX-A12	Macugen/Pegaptanib
**Aptamer type**	Unmodified guanosine rich DNA	RNA Spiegelmer	PEGylated RNA with 2′-F and 2′-OMe modifications
**Generation method**	Chemically synthesized consisting entirely of deoxyguanosine and thymidine.	SELEX is carried out against a mirror-image target, an aptamer recognizing the mirrored target is generated. Therefore, the corresponding mirror image configuration of the generated aptamer, L-aptamer/spiegelmer, recognizes the natural target.	Protein SELEX was carried out, only involving positive selection with recombinant VEGF_165_.
**Target**	Cell surface Nucleolin (NCL)	Stromal cell-derived factor 1 (SDF1/CXCL12)	Vascular endothelial growth factor 165 (VEGF_165_)
**Mechanism of action**	Receptor mediated internalization and inhibition of DNA replication *via* NCL	Upstream pathway inhibition through binding to SDF1	Direct extracellular signal inhibition through binding to VEGF_165_
**References**	(Bates et al., 1999; Bates et al., 2009)	(Eulberg and Klussmann, 2003; Hoellenriegel et al., 2014; Roccaro Aldo et al., 2014)	(Jellinek et al., 1994; Ng et al., 2006; Xie et al., 2019)

The most well understood example of successful therapeutic aptamers is Macugen, an RNA aptamer which is applied intraocularly to inhibit age-related macular degeneration through isoform specific competitive binding to VEGF. Macugen is the only aptamer to date with FDA approval for therapeutic use (Mercier et al., 2017; Hori et al., 2018). Despite Macugen’s minimal side effects and therapeutic efficacy it is rarely used in clinical practice due to development of more efficient treatment options that have higher binding affinity and efficacy (Klein and Liang, 2018). Despite its retirement from treatment of age-related macular degeneration, the Macugen aptamer’s ability to inhibit aberrant VEGF mediated vascularization and angiogenesis makes it an interesting therapeutic option under investigation for a range of other diseases including cancer (Bunka et al., 2010).

Another successful aptamer therapeutic is NOX-A12, a spiegelmer—an unnatural mirror image nucleic acid aptamer strand—which targets CXCL12/SDF-1, a key chemokine involved in the maintenance of the tumor microenvironment in chronic lymphocytic leukemia and multiple myeloma cancers (Hoellenriegel et al., 2014; Roccaro Aldo et al., 2014). When NOX-A12 is bound to CXCL12 the binding inhibits the development of a CXCL12 gradient in basement membrane stromal cells of the tumor microenvironment, which disrupts tumor proliferation and metastatic ability, and diminishes drug resistance in tumor cells (Kaur et al., 2018). This aptamer has been shown to be well tolerated in patients with minimal adverse events occurring in several stage I/II clinical trials and has been granted orphan drug designation by the FDA for glioblastoma treatment with radiotherapy (Kaur et al., 2018). The successes of NOX-A12 positions this aptamer as a potent anti-cancer drug.

A second aptamer that has been investigated in several stage I/II clinical trials is AS1411 which binds to nucleolin on the cell surface and has been shown to reduce cell proliferation though induction of apoptosis (Wu et al., 2017). AS1411 was the first aptamer to enter human clinical trials for cancer therapy, though phase II trials have shown minimal overall efficacy in metastatic refractory renal cell carcinoma patients (Rosenberg et al., 2014). Despite this, AS1411’s broad cancer targeting in numerous subtypes (Chen et al., 2007; Cho et al., 2016; Carvalho et al., 2019) and functional anticancer role upon internalization remains encouraging for its development as a drug-free therapeutic agent. In addition, the mechanism of binding and internalization of AS1411 is particularly interesting as it does not internalize by standard receptor mediated endocytosis (Reyes-Reyes et al., 2015), instead it is internalized by micropinocytosis and released, rather than recycled or degraded, from endosomal compartments *via* a non-standard mechanism. This allows the nucleolin targeting AS1411 aptamers to translocate between the nucleus and cytoplasm of target cells affording efficient intracellular delivery of conjugated drugs (Kotula et al., 2012). AS1411 therefore represents the gold standard of an internalizing aptamer targeting intracellular compartments for anti-cancer therapeutics, as most issues in clinical translation of therapeutic aptamers relate to their susceptibility to endosomal degradation, nuclease degradation, or rapid renal clearance (Sundaram et al., 2013; Wu et al., 2017).

Together these aptamers demonstrate the extent to which aptamer technology has evolved, from simple diagnostic tools to fully functional FDA approved therapeutics and all stages between for a range of therapeutic applications, of which cancer is particularly promising.

## Final Thoughts

The delivery of anti-cancer therapeutics to tumor cells has long been difficult to do in practice, due to size, charge, and uptake restrictions (Wagstaff and Jans, 2009). DNA aptamer mediated cell-targeting and internalization can overcome most of these issues, aided by their small size and highly efficient binding and cellular uptake mechanisms. This aspect alone cements aptamers as a robust class of targeting agents with ability to deliver therapeutics to intracellular targets and compartments. It is encouraging furthermore to see that aptamers are also capable of functional activity upon binding and can act independently as drug-free therapeutics.

Nonetheless, aptamer technology still has a few obstacles to overcome before they can be more widely accepted as therapeutics for cancer. A significant hurdle in the development of therapeutic aptamers is clinical translation. Due to difficulties regarding *in vivo* targeting specificity, susceptibility to nuclease degradation unless modified, which may result in unwanted toxicity and difficulty identifying the affected intracellular pathways following internalization, aptamers making it to the later clinical trial stages are scarce.

Approaches such as *in vivo* SELEX and cell-SELEX are beginning to address the issues of *in vivo* stability and targeting ability through stringent selection rounds and in-depth characterization of serum stability and binding affinity, followed by imaging in cells and animal models using fluorescently conjugated aptamers to determine where aptamers are localized following internalization (Bouvier-Müller and Ducongé, 2018). Much is still unknown however about how they affect their targets within the cell due to the complexity in characterizing their targets, their internalization methods, and furthermore knowing what functional anti-cancer activity to measure. Deeper understanding of an aptamer’s molecular target is often required to be able to first hypothesize and then test what functional changes may happen upon aptamer binding and internalization into target cells/tumors/tissues. While many of the studies cited in this review had determined specific targets of the aptamers in question, perhaps the largest challenge of aptamer generation by cell-SELEX is still target identification (Mercier et al., 2017). Cell membrane associated proteins are notoriously difficult to isolate, and the targets of aptamers are not always the most abundant protein of the sample, making downstream proteomic identification approaches difficult. While there are some advances in the approaches towards aptamer target identification using chromatographic approaches (Drabik et al., 2018) or by detailed proteomic analysis (Hou et al., 2015), most approaches rely upon effective pulldown using the aptamer and subsequent mass spectrometry of bound proteins. Other interesting approaches to target identification involve direct binding assays akin to traditional ELISA techniques with aptamers substituting for antibodies (Toh et al., 2015) or a western blot-like technique involving aptamer mediated probing of immobilized proteins dubbed “southwestern blotting” (Bates et al., 2009; Nastasijevic et al., 2012).

Another difficulty in developing aptamer therapeutics is following cell internalization, aptamers must have efficient endosomal escape ability thereby attaining effective intracellular delivery. This can be limited by target function, but also may be impacted by aptamer stability *in vivo*. This difficulty in attaining robust internalizing aptamers has led to the development of modified SELEX approaches, which only select for internalized sequences during the initial aptamer generation process. Beyond the scope of this review other approaches using nanoparticles have also been shown to increase internalization capacity (Zhang et al., 2013) and enable endosomal escape through specific molecular degradation of the endosome delivered by conjugated molecules (Ma, 2014; Sun et al., 2014; Xiang et al., 2015). Furthermore, chemical modification of the nucleic acid sugar backbones or inclusion of unnatural bases into the aptamer (Ni et al., 2017) and use of spiegelmers (Eulberg and Klussmann, 2003) to generate more stable/nuclease resistant aptamers has shown to be effective in overcoming stability and internalization issues associated with using aptamers as intracellular therapeutics.

Through novel SELEX procedures enabling new guided targeting abilities, different target identification strategies and the relative ease of chemical modification, aptamer generation is becoming ever more advanced. What once was thought to be a “magic bullet” type therapeutic seems to be materializing in the form of aptamers. They represent highly specific targeting agents, with inbuilt capability to evoke cytostatic effects upon their targets, while minimizing damage to surrounding healthy tissue. The use of aptamers as unconjugated therapeutics is entirely possible but will rely upon innovation and dedication to appropriately characterize their functions *in vivo* for intracellular targeting before they will be at a standard capable of entering into widespread clinical use.

## Author Contributions

MM drafted the initial manuscript and made the figures/tables. KW had the initial idea for the review and the created outline of what to include and extensively edited the drafts.

## Funding

KW is supported by a Career Development Fellowship from the National Breast Cancer Foundation (ECF-17-007). MM is supported by an RTP Scholarship from the Australian Department of Education, Skills and Employment and a health and biosecurity top-up scholarship from the Commonwealth Scientific and Industrial Research Organisation.

## Conflict of Interest

The authors declare that the research was conducted in the absence of any commercial or financial relationships that could be construed as a potential conflict of interest.

## References

[B1] Abnous K. Danesh N. M. Ramezani M. Yazdian-Robati R. Alibolandi M. Taghdisi S. M. (2017). A novel chemotherapy drug-free delivery system composed of three therapeutic aptamers for the treatment of prostate and breast cancers in vitro and in vivo. Nanomed. Nanotechnol. Biol. Med. 13 (6), 1933–1940. 10.1016/j.nano.2017.04.002 28414074

[B2] Ali M. H. Elsherbiny M. E. Emara M. (2019). Updates on Aptamer Research. Int. J. Mol. Sci. 20 (10), 2511. 10.3390/ijms20102511 PMC6566374 31117311

[B3] Amadio M. Govoni S. Pascale A. (2016). Targeting VEGF in eye neovascularization: What’s new?: A comprehensive review on current therapies and oligonucleotide-based interventions under development. Pharmacol. Res. 103, 253–269. 10.1016/j.phrs.2015.11.027 26678602

[B4] Aptekar S. Arora M. Lawrence C. L. Lea R. W. Ashton K. Dawson T. (2015). Selective Targeting to Glioma with Nucleic Acid Aptamers. PloS One 10 (8), e0134957. 10.1371/journal.pone.0134957 26252900 PMC4529171

[B5] Banerjee J. Nilsen-Hamilton M. (2013). Aptamers: multifunctional molecules for biomedical research. J. Mol. Med. 91 (12), 1333–1342. 10.1007/s00109-013-1085-2 24045702

[B6] Baskar R. Lee K. A. Yeo R. Yeoh K.-W. (2012). Cancer and radiation therapy: current advances and future directions. Int. J. Med. Sci. 9 (3), 193–199. 10.7150/ijms.3635 22408567 PMC3298009

[B7] Bates P. J. Kahlon J. B. Thomas S. D. Trent J. O. Miller D. M. (1999). Antiproliferative activity of G-rich oligonucleotides correlates with protein binding. J. Biol. Chem. 274 (37), 26369–26377. 10.1074/jbc.274.37.26369 10473594

[B8] Bates P. J. Laber D. A. Miller D. M. Thomas S. D. Trent J. O. (2009). Discovery and development of the G-rich oligonucleotide AS1411 as a novel treatment for cancer. Exp. Mol. Pathol. 86 (3), 151–164. 10.1016/j.yexmp.2009.01.004 19454272 PMC2716701

[B9] Benedetto G. Hamp T. J. Wesselman P. J. Richardson C. (2015). Identification of epithelial ovarian tumor-specific aptamers. Nucleic Acid Ther. 25 (3), 162–172. 10.1089/nat.2014.0522 25894736 PMC4440997

[B10] Bengtsson E. Malm P. (2014). Screening for Cervical Cancer Using Automated Analysis of PAP-Smears. Comput. Math. Methods Med. 2014:842037. 10.1155/2014/842037 24772188 PMC3977449

[B11] Blake C. M. Sullenger B. A. Lawrence D. A. Fortenberry Y. M. (2009). Antimetastatic potential of PAI-1-specific RNA aptamers. Oligonucleotides 19 (2), 117–128. 10.1089/oli.2008.0177 19284310 PMC2948460

[B12] Bouvier-Müller A. Ducongé F. (2018). Application of aptamers for in vivo molecular imaging and theranostics. Adv. Drug Deliv. Rev. 134, 94–106. 10.1016/j.addr.2018.08.004 30125606

[B13] Bunka D. H. Platonova O. Stockley P. G. (2010). Development of aptamer therapeutics. Curr. Opin. Pharmacol. 10 (5), 557–562. 10.1016/j.coph.2010.06.009 20638902

[B14] Camorani S. Crescenzi E. Gramanzini M. Fedele M. Zannetti A. Cerchia L. (2017). Aptamer-mediated impairment of EGFR-integrin αvβ3 complex inhibits vasculogenic mimicry and growth of triple-negative breast cancers. Sci. Rep. 7, 46659–. 10.1038/srep46659 28425453 PMC5397976

[B15] Carvalho J. Paiva A. Cabral Campello M. P. Paulo A. Mergny J.-L. Salgado G. F. (2019). Aptamer-based Targeted Delivery of a G-quadruplex Ligand in Cervical Cancer Cells. Sci. Rep. 9 (1), 7945. 10.1038/s41598-019-44388-9 31138870 PMC6538641

[B16] Catuogno S. Esposito C. L. (2017). Aptamer Cell-Based Selection: Overview and Advances. Biomedicines 5 (3), 49. 10.3390/biomedicines5030049 PMC5618307 28805744

[B17] Catuogno S. Esposito C. L. de Franciscis V. (2016). Aptamer-Mediated Targeted Delivery of Therapeutics: An Update. Pharmaceuticals 9 (4), 69. 10.3390/ph9040069 PMC5198044 27827876

[B18] Cerchia L. de Franciscis V. (2010). Targeting cancer cells with nucleic acid aptamers. Trends Biotechnol. 28 (10), 517–525. 10.1016/j.tibtech.2010.07.005 20719399

[B19] Chandrasekaran R. Lee A. S. Yap L. W. Jans D. A. Wagstaff K. M. Cheng W. (2016). Tumor cell-specific photothermal killing by SELEX-derived DNA aptamer-targeted gold nanorods. Nanoscale 8 (1), 187–196. 10.1039/c5nr07831h 26646051

[B20] Chen W. Sridharan V. Soundararajan S. Otake Y. Stuart R. Jones D. (2007). Activity and Mechanism of Action of AS1411 in Acute Myeloid Leukemia Cells. Blood 110 (11), 1604–. 10.1182/blood.V110.11.1604.1604

[B21] Chen M. Yu Y. Jiang F. Zhou J. Li Y. Liang C. (2016). Development of Cell-SELEX Technology and Its Application in Cancer Diagnosis and Therapy. Int. J. Mol. Sci. 17 (12), 2079. 10.3390/ijms17122079 PMC5187879 27973403

[B22] Chen L. He W. Jiang H. Wu L. Xiong W. Li B. (2019). In vivo SELEX of bone targeting aptamer in prostate cancer bone metastasis model. Int. J. Nanomed. 14, 149–159. 10.2147/IJN.S188003 PMC6306056 30613143

[B23] Cho Y. Lee Y. B. Lee J.-H. Lee D. H. Cho E. J. Yu S. J. (2016). Modified AS1411 Aptamer Suppresses Hepatocellular Carcinoma by Up-Regulating Galectin-14. PloS One 11 (8), e0160822. 10.1371/journal.pone.0160822 27494117 PMC4975508

[B24] Dassie J. P. Liu X. Y. Thomas G. S. Whitaker R. M. Thiel K. W. Stockdale K. R. (2009). Systemic administration of optimized aptamer-siRNA chimeras promotes regression of PSMA-expressing tumors. Nat. Biotechnol. 27 (9), 839–849. 10.1038/nbt.1560 19701187 PMC2791695

[B25] Deng B. Lin Y. Wang C. Li F. Wang Z. Zhang H. (2014). Aptamer binding assays for proteins: the thrombin example–a review. Anal. Chim. Acta 837, 1–15. 10.1016/j.aca.2014.04.055 25000852

[B26] Dillman R. O. (1999). Perceptions of Herceptin®: A Monoclonal Antibody for the Treatment of Breast Cancer. Cancer Biother. Radiopharmaceut. 14 (1), 5–10. 10.1089/cbr.1999.14.5 10850281

[B27] Drabik A. Ner-Kluza J. Mielczarek P. Civit L. Mayer G. Silberring J. (2018). Advances in the Study of Aptamer–Protein Target Identification Using the Chromatographic Approach. J. Proteome Res. 17 (6), 2174–2181. 10.1021/acs.jproteome.8b00122 29703078

[B28] Dua P. Kim S. (2011). Lee D-k. Nucleic acid aptamers targeting cell-surface proteins. Methods 54 (2), 215–225. 10.1016/j.ymeth.2011.02.002 21300154

[B29] Duan M. Long Y. Yang C. Wu X. Sun Y. Li J. (2016). Selection and characterization of DNA aptamer for metastatic prostate cancer recognition and tissue imaging. Oncotarget 7 (24), 36436–36446. 10.18632/oncotarget.9262 27183906 PMC5095011

[B30] Ellington A. D. Szostak J. W. (1990). In vitro selection of RNA molecules that bind specific ligands. Nature 346 (6287), 818–822. 10.1038/346818a0 1697402

[B31] Esposito C. L. Passaro D. Longobardo I. Condorelli G. Marotta P. Affuso A. (2011). A neutralizing RNA aptamer against EGFR causes selective apoptotic cell death. PloS One 6 (9), e24071–e2407e. 10.1371/journal.pone.0024071 21915281 PMC3167817

[B32] Eulberg D. Klussmann S. (2003). Spiegelmers: biostable aptamers. Chembiochem 4 (10), 979–983. 10.1002/cbic.200300663 14523914

[B33] Ferlay J. Colombet M. Soerjomataram I. Mathers C. Parkin D. M. Piñeros M. (2019). Estimating the global cancer incidence and mortality in 2018: GLOBOCAN sources and methods. Int. J. Cancer 144 (8), 1941–1953. 10.1002/ijc.31937 30350310

[B34] Fortenberry Y. M. Brandal S. M. Carpentier G. Hemani M. Pathak A. P. (2016). Intracellular Expression of PAI-1 Specific Aptamers Alters Breast Cancer Cell Migration, Invasion and Angiogenesis. PloS One 11 (10), e0164288. 10.1371/journal.pone.0164288 27755560 PMC5068744

[B35] Gomes de Castro M. A. Höbartner C. Opazo F. (2017). Aptamers provide superior stainings of cellular receptors studied under super-resolution microscopy. PloS One 12 (2), e0173050. 10.1371/journal.pone.0173050 28235049 PMC5325610

[B36] Hicke B. J. Marion C. Chang Y. F. Gould T. Lynott C. K. Parma D. (2001). Tenascin-C aptamers are generated using tumor cells and purified protein. J. Biol. Chem. 276 (52), 48644–48654. 10.1074/jbc.M104651200 11590140

[B37] Hoellenriegel J. Zboralski D. Maasch C. Rosin N. Y. Wierda W. G. Keating M. J. (2014). The Spiegelmer NOX-A12, a novel CXCL12 inhibitor, interferes with chronic lymphocytic leukemia cell motility and causes chemosensitization. Blood 123 (7), 1032–1039. 10.1182/blood-2013-03-493924 24277076 PMC4123413

[B38] Hori S. I. Herrera A. Rossi J. J. Zhou J. (2018). Current Advances in Aptamers for Cancer Diagnosis and Therapy. Cancers (Basel) 10 (1). 10.3390/cancers10010009 PMC5789359 29301363

[B39] Hossain S. Chowdhury E. H. Akaike T. (2011). Nanoparticles and toxicity in therapeutic delivery: the ongoing debate. Ther. Deliv. 2 (2), 125–132. 10.4155/tde.10.109 22833937

[B40] Hou Z. Meyer S. Propson N. E. Nie J. Jiang P. Stewart R. (2015). Characterization and target identification of a DNA aptamer that labels pluripotent stem cells. Cell Res. 25 (3), 390–393. 10.1038/cr.2015.7 25591927 PMC4349250

[B41] Hu Y. Duan J. Zhan Q. Wang F. Lu X. Yang X. D. (2012). Novel MUC1 aptamer selectively delivers cytotoxic agent to cancer cells in vitro. PloS One 7 (2), e31970. 10.1371/journal.pone.0031970 22384115 PMC3284512

[B42] Hung L. Y. Wang C. H. Hsu K. F. Chou C. Y. Lee G. B. (2014). An on-chip Cell-SELEX process for automatic selection of high-affinity aptamers specific to different histologically classified ovarian cancer cells. Lab. Chip. 14 (20), 4017–4028. 10.1039/c4lc00587b 25144781

[B43] Iaboni M. Fontanella R. Rienzo A. Capuozzo M. Nuzzo S. Santamaria G. (2016). Targeting Insulin Receptor with a Novel Internalizing Aptamer. Mol. Ther. Nucleic Acids 5 (9), e365–e36e. 10.1038/mtna.2016.73 27648925 PMC5056995

[B44] Jellinek D. Green L. S. Bell C. Janjic N. (1994). Inhibition of receptor binding by high-affinity RNA ligands to vascular endothelial growth factor. Biochemistry 33 (34), 10450–10456. 10.1021/bi00200a028 7520755

[B45] Jenison R. D. Gill S. C. Pardi A. Polisky B. (1994). High-resolution molecular discrimination by RNA. Sci. (N. Y. NY) 263 (5152), 1425–1429. 10.1126/science.7510417 7510417

[B46] Kaur H. Bruno J. G. Kumar A. Sharma T. K. (2018). Aptamers in the Therapeutics and Diagnostics Pipelines. Theranostics 8 (15), 4016–4032. 10.7150/thno.25958 30128033 PMC6096388

[B47] Keefe A. D. Pai S. Ellington A. (2010). Aptamers as therapeutics. Nat. Rev. Drug Discovery 9 (7), 537–550. 10.1038/nrd3141 20592747 PMC7097324

[B48] Kim M. Kim D.-M. Kim K.-S. Jung W. Kim D.-E. (2018). Applications of Cancer Cell-Specific Aptamers in Targeted Delivery of Anticancer Therapeutic Agents. Molecules 23 (4), 830. 10.3390/molecules23040830 PMC6017884 29617327

[B49] Klein K. Liang M. C. (2018). “Chapter 7 - Anti–Vascular Endothelial Growth Factor Therapy for Diabetic Eye Disease,” in Current Management of Diabetic Retinopathy. Eds. Baumal C. R. Duker J. S. (St. Louis, Missouri: Elsevier), 53–78.

[B50] Komarova N. Kuznetsov A. (2019). Inside the Black Box: What Makes SELEX Better? Molecules 24 (19), 3598. 10.3390/molecules24193598 PMC6804172 31591283

[B51] Kong H. Y. Byun J. (2013). Nucleic Acid aptamers: new methods for selection, stabilization, and application in biomedical science. Biomol. Ther. 21 (6), 423–434. 10.4062/biomolther.2013.085 PMC3879913 24404332

[B52] Kotula J. W. Pratico E. D. Ming X. Nakagawa O. Juliano R. L. Sullenger B. A. (2012). Aptamer-mediated delivery of splice-switching oligonucleotides to the nuclei of cancer cells. Nucleic Acid Ther. 22 (3), 187–195. 10.1089/nat.2012.0347 22703281 PMC3423875

[B53] Lee J.-H. Canny M. D. De Erkenez A. Krilleke D. Ng Y.-S. Shima D. T. (2005). A therapeutic aptamer inhibits angiogenesis by specifically targeting the heparin binding domain of VEGF_165_ . Proc. Natl. Acad. Sci. U. States America 102 (52), 18902. 10.1073/pnas.0509069102 PMC1323181 16357200

[B54] Li H. Yang S. Yu G. Shen L. Fan J. Xu L. (2017). Aptamer Internalization via Endocytosis Inducing S-Phase Arrest and Priming Maver-1 Lymphoma Cells for Cytarabine Chemotherapy. Theranostics 7 (5), 1204–1213. 10.7150/thno.17069 28435459 PMC5399587

[B55] Liu M. Yang T. Chen Z. Wang Z. He N. (2018). Differentiating breast cancer molecular subtypes using a DNA aptamer selected against MCF-7 cells. Biomater. Sci. 6 (12), 3152–3159. 10.1039/C8BM00787J 30349922

[B56] Ma D. (2014). Enhancing endosomal escape for nanoparticle mediated siRNA delivery. Nanoscale 6 (12), 6415–6425. 10.1039/C4NR00018H 24837409

[B57] Mallikaratchy P. (2017). Evolution of Complex Target SELEX to Identify Aptamers against Mammalian Cell-Surface Antigens. Molecules 22 (2), 215. 10.3390/molecules22020215 PMC5572134 28146093

[B58] Mascini M. Palchetti I. Tombelli S. (2012). Nucleic Acid and Peptide Aptamers: Fundamentals and Bioanalytical Aspects. Angewandte Chem. Int. Ed. 51 (6), 1316–1332. 10.1002/anie.201006630 22213382

[B59] McKeague M. Derosa M. C. (2012). Challenges and opportunities for small molecule aptamer development. J. Nucleic Acids 2012, 748913–. 10.1155/2012/748913 23150810 PMC3488411

[B60] Mercier M.-C. Dontenwill M. Choulier L. (2017). Selection of Nucleic Acid Aptamers Targeting Tumor Cell-Surface Protein Biomarkers. Cancers (Basel) 9 (6), 69. 10.3390/cancers9060069 PMC5483888 28635657

[B61] Mittal S. Kaur H. Gautam N. Mantha A. K. (2017). Biosensors for breast cancer diagnosis: A review of bioreceptors, biotransducers and signal amplification strategies. Biosens Bioelectron 88, 217–231. 10.1016/j.bios.2016.08.028 27567264

[B62] Molina M. A. Codony-Servat J. Albanell J. Rojo F. Arribas J. Baselga J. (2001). Trastuzumab (Herceptin), a Humanized Anti-HER2 Receptor Monoclonal Antibody, Inhibits Basal and Activated HER2 Ectodomain Cleavage in Breast Cancer Cells. Cancer Res. 61 (12), 4744. 11406546

[B63] Nastasijevic B. Wright B. R. Smestad J. Warrington A. E. Rodriguez M. Maher L. J. (2012). III. Remyelination Induced by a DNA Aptamer in a Mouse Model of Multiple Sclerosis. PloS One 7 (6), e39595. 10.1371/journal.pone.0039595 22761835 PMC3384608

[B64] Ng E. W. M. Shima D. T. Calias P. Cunningham E. T. Guyer D. R. Adamis A. P. (2006). Pegaptanib, a targeted anti-VEGF aptamer for ocular vascular disease. Nat. Rev. Drug Discovery 5 (2), 123–132. 10.1038/nrd1955 16518379

[B65] Ni S. Yao H. Wang L. Lu J. Jiang F. Lu A. (2017). Chemical Modifications of Nucleic Acid Aptamers for Therapeutic Purposes. Int. J. Mol. Sci. 18 (8), 1683. 10.3390/ijms18081683 PMC5578073 28767098

[B66] Odeh F. Nsairat H. Alshaer W. Ismail M. A. Esawi E. Qaqish B. (2019). Aptamers Chemistry: Chemical Modifications and Conjugation Strategies. Molecules 25 (1), 3. 10.3390/molecules25010003 PMC6982925 31861277

[B67] Pang X. Cui C. Wan S. Jiang Y. Zhang L. Xia L. (2018). Bioapplications of Cell-SELEX-Generated Aptamers in Cancer Diagnostics, Therapeutics, Theranostics and Biomarker Discovery: A Comprehensive Review. Cancers (Basel) 10 (2), 47. 10.3390/cancers10020047 PMC5836079 29425173

[B68] Patra J. K. Das G. Fraceto L. F. Campos E. V. R. MdP R.-T. LS A.-T. (2018). Nano based drug delivery systems: recent developments and future prospects. J. Nanobiotechnol. 16 (1), 71. 10.1186/s12951-018-0392-8 PMC6145203 30231877

[B69] Pereira R. L. Nascimento I. C. Santos A. P. Ogusuku I. E. Y. Lameu C. Mayer G. (2018). Aptamers: novelty tools for cancer biology. Oncotarget 9 (42), 26934–26953. 10.18632/oncotarget.25260 29928493 PMC6003562

[B70] Reyes-Reyes E. M. Šalipur F. R. Shams M. Forsthoefel M. K. Bates P. J. (2015). Mechanistic studies of anticancer aptamer AS1411 reveal a novel role for nucleolin in regulating Rac1 activation. Mol. Oncol. 9 (7), 1392–1405. 10.1016/j.molonc.2015.03.012 25911416 PMC4523413

[B71] Roccaro Aldo M. Sacco A. Purschke Werner G. Moschetta M. Buchner K. Maasch C. (2014). SDF-1 Inhibition Targets the Bone Marrow Niche for Cancer Therapy. Cell Rep. 9 (1), 118–128. 10.1016/j.celrep.2014.08.042 25263552 PMC4194173

[B72] Rosenberg J. E. Bambury R. M. Van Allen E. M. Drabkin H. A. Lara P. N. Jr. Harzstark A. L. (2014). A phase II trial of AS1411 (a novel nucleolin-targeted DNA aptamer) in metastatic renal cell carcinoma. Invest. New Drugs 32 (1), 178–187. 10.1007/s10637-013-0045-6 24242861 PMC4560460

[B73] Röthlisberger P. Hollenstein M. (2018). Aptamer chemistry. Adv. Drug Deliv. Rev. 134, 3–21. 10.1016/j.addr.2018.04.007 29626546

[B74] Ruckman J. Green L. S. Beeson J. Waugh S. Gillette W. L. Henninger D. D. (1998). 2’-Fluoropyrimidine RNA-based aptamers to the 165-amino acid form of vascular endothelial growth factor (VEGF165). Inhibition of receptor binding and VEGF-induced vascular permeability through interactions requiring the exon 7-encoded domain. J. Biol. Chem. 273 (32), 20556–20567. 10.1074/jbc.273.32.20556 9685413

[B75] Sekhon N. Kumbla R. A. Mita M. (2017). “Chapter 1 - Current Trends in Cancer Therapy,” in Cardio-Oncology. Eds. Gottlieb R. A. Mehta P. K. (Boston: Academic Press), 1–24.

[B76] Shangguan D. Li Y. Tang Z. Cao Z. C. Chen H. W. Mallikaratchy P. (2006). Aptamers evolved from live cells as effective molecular probes for cancer study. Proc. Natl. Acad. Sci. 103 (32), 11838–11843. 10.1073/pnas.0602615103 16873550 PMC1567664

[B77] Sun H. Zhu X. Lu P. Y. Rosato R. R. Tan W. Zu Y. (2014). Oligonucleotide aptamers: new tools for targeted cancer therapy. Mol. Ther. Nucleic Acids 3, e182. 10.1038/mtna.2014.32 25093706 PMC4221593

[B78] Sundaram P. Kurniawan H. Byrne M. E. Wower J. (2013). Therapeutic RNA aptamers in clinical trials. Eur. J. Pharm. Sci. 48 (1), 259–271. 10.1016/j.ejps.2012.10.014 23142634

[B79] Tabarzad M. Jafari M. (2016). Trends in the Design and Development of Specific Aptamers Against Peptides and Proteins. Protein J. 35 (2), 81–99. 10.1007/s10930-016-9653-2 26984473

[B80] Talbot L. J. Mi Z. Bhattacharya S. D. Kim V. Guo H. Kuo P. C. (2011). Pharmacokinetic characterization of an RNA aptamer against osteopontin and demonstration of in vivo efficacy in reversing growth of human breast cancer cells. Surgery 150 (2), 224–230. 10.1016/j.surg.2011.05.015 21801960 PMC3148491

[B81] Thiel W. H. Thiel K. W. Flenker K. S. Bair T. Dupuy A. J. McNamara J. O., 2. (2015). Cell-internalization SELEX: method for identifying cell-internalizing RNA aptamers for delivering siRNAs to target cells. Methods Mol. Biol. 1218, 187–199. 10.1007/978-1-4939-1538-5_11 25319652 PMC4435695

[B82] Toh S. Y. Citartan M. Gopinath S. C. Tang T. H. (2015). Aptamers as a replacement for antibodies in enzyme-linked immunosorbent assay. Biosens Bioelectron 64, 392–403. 10.1016/j.bios.2014.09.026 25278480

[B83] Tuerk C. Gold L. (1990). Systematic evolution of ligands by exponential enrichment: RNA ligands to bacteriophage T4 DNA polymerase. Sci. (N. Y. NY) 249 (4968), 505–510. 10.1126/science.2200121 2200121

[B84] Van Simaeys D. Turek D. Champanhac C. Vaizer J. Sefah K. Zhen J. (2014). Identification of cell membrane protein stress-induced phosphoprotein 1 as a potential ovarian cancer biomarker using aptamers selected by cell systematic evolution of ligands by exponential enrichment. Anal. Chem. 86 (9), 4521–4527. 10.1021/ac500466x 24654750 PMC4018121

[B85] Wagstaff K. M. Jans D. A. (2009). Nuclear drug delivery to target tumour cells. Eur. J. Pharmacol. 625 (1-3), 174–180. 10.1016/j.ejphar.2009.06.069 19836384

[B86] Wang R. E. Wu H. Niu Y. Cai J. (2011a). Improving the stability of aptamers by chemical modification. Curr. Med. Chem. 18 (27), 4126–4138. 10.2174/092986711797189565 21838692

[B87] Wang R. E. Zhang Y. Cai J. Cai W. Gao T. (2011b). Aptamer-based fluorescent biosensors. Curr. Med. Chem. 18 (27), 4175–4184. 10.2174/092986711797189637 21838688 PMC3205236

[B88] Wu X. Shaikh A. B. Yu Y. Li Y. Ni S. Lu A. (2017). Potential Diagnostic and Therapeutic Applications of Oligonucleotide Aptamers in Breast Cancer. Int. J. Mol. Sci. 18 (9), 1851. 10.3390/ijms18091851 PMC5618500 28841163

[B89] Xiang D. Shigdar S. Qiao G. Wang T. Kouzani A. Z. Zhou S.-F. (2015). Nucleic Acid Aptamer-Guided Cancer Therapeutics and Diagnostics: the Next Generation of Cancer Medicine. Theranostics 5 (1), 23–42. 10.7150/thno.10202 25553096 PMC4265746

[B90] Xie X. Zhang Y. Ma W. Shao X. Zhan Y. Mao C. (2019). Potent anti-angiogenesis and anti-tumour activity of pegaptanib-loaded tetrahedral DNA nanostructure. Cell Proliferation 52 (5), e12662. 10.1111/cpr.12662 31364793 PMC6797503

[B91] Yang X. Li N. Gorenstein D. G. (2011). Strategies for the discovery of therapeutic aptamers. Expert Opin. Drug Discovery 6 (1), 75–87. 10.1517/17460441.2011.537321 PMC3045091 21359096

[B92] Yildizhan H. Barkan N. P. Karahisar Turan S. Demiralp Ö Özel Demiralp F. D. Uslu B. (2018). “Chapter 1 - Treatment strategies in cancer from past to present,” in Drug Targeting and Stimuli Sensitive Drug Delivery Systems. Ed. Grumezescu A. M. (Norwich, United States: William Andrew Publishing), 1–37.

[B93] Yoon S. Rossi J. J. (2018). Aptamers: Uptake mechanisms and intracellular applications. Adv. Drug Deliv. Rev. 134, 22–35. 10.1016/j.addr.2018.07.003 29981799 PMC7126894

[B94] Yoon S. Armstrong B. Habib N. Rossi J. J. (2017). Blind SELEX Approach Identifies RNA Aptamers That Regulate EMT and Inhibit Metastasis. Mol. Cancer Res. MCR 15 (7), 811–820. 10.1158/1541-7786.MCR-16-0462 28396463 PMC5555160

[B95] Zhang J. Liu B. Liu H. Zhang X. Tan W. (2013). Aptamer-conjugated gold nanoparticles for bioanalysis. Nanomed. (Lond) 8 (6), 983–993. 10.2217/nnm.13.80 23730697

[B96] Zhao F. Zhou J. Su X. Wang Y. Yan X. Jia S. (2017). A Smart Responsive Dual Aptamers-Targeted Bubble-Generating Nanosystem for Cancer Triplex Therapy and Ultrasound Imaging. Small (Weinheim Der Bergstrasse Germany) 13 (20). 10.1002/smll.201603990 28371376

[B97] Zhou J. Rossi J. (2017). Aptamers as targeted therapeutics: current potential and challenges. Nat. Rev. Drug Discovery 16 (3), 181–202. 10.1038/nrd.2016.199 27807347 PMC5700751

[B98] Zhu H. Li J. Zhang X. B. Ye M. Tan W. (2015). Nucleic acid aptamer-mediated drug delivery for targeted cancer therapy. ChemMedChem 10 (1), 39–45. 10.1002/cmdc.201402312 25277749

[B99] Zhu Q. Liu G. Kai M. (2015). DNA Aptamers in the Diagnosis and Treatment of Human Diseases. Molecules 20 (12), 20979–20997. 10.3390/molecules201219739 26610462 PMC6332121

[B100] Zhu G. Zhang H. Jacobson O. Wang Z. Chen H. Yang X. (2017). Combinatorial Screening of DNA Aptamers for Molecular Imaging of HER2 in Cancer. Bioconjugate Chem. 28 (4), 1068–1075. 10.1021/acs.bioconjchem.6b00746 28122449

[B101] Zou Y. Duan N. Wu S. Shen M. Wang Z. (2018). Selection, Identification, and Binding Mechanism Studies of an ssDNA Aptamer Targeted to Different Stages of E. coli O157:H7. J. Agric. Food Chem. 66 (22), 5677–5682. 10.1021/acs.jafc.8b01006 29756774

